# Microwave Assisted Convenient One-Pot Synthesis of Coumarin Derivatives via Pechmann Condensation Catalyzed by FeF_3_ under Solvent-Free Conditions and Antimicrobial Activities of the Products

**DOI:** 10.3390/molecules190913093

**Published:** 2014-08-26

**Authors:** Vahid Vahabi, Farhad Hatamjafari

**Affiliations:** Department of Chemistry, Faculty of Science, Islamic Azad University-Tonekabon Branch, Tonekabon 46841-61167, Iran; E-Mail: f_hatamjafari@tonekaboniau.ac.ir

**Keywords:** coumarin, solvent-free, FeF3, one-pot, microwave irradiation, antimicrobial activities

## Abstract

A rapid and efficient solvent-free one-pot synthesis of coumarin derivatives by Pechmann condensation reactions of phenols with ethyl acetoacetate using FeF_3_ as a catalyst under microwave irradiation is described. This one-pot synthesis on a solid inorganic support provides the products in good yields. The newly synthesized compounds were systematically characterized by IR, ^1^H-NMR, ^13^C-NMR, MS and elemental CHN analyses. The proposed solvent-free microwave irradiation method using the environmentally friendly catalyst FeF_3_ offers the unique advantages of high yields, shorter reaction times, easy and quick isolation of the products, excellent chemoselectivity, and a one-pot, green synthesis. The products were screened for antimicrobial activity, and the results showed that the compounds reacted against all the tested bacteria.

## 1. Introduction

Coumarin and its derivatives are biologically and pharmacologically active compounds with a wide range of properties as antitumor, antimicrobial, anti-HIV, anticoagulant, anti-inflammatory and antioxidant agents [1,2]. In particular, the antitumor activity of coumarin compounds has received considerable attention among researchers. Coumarins belong to the flavonoid class of compounds that are mainly isolated from natural plants. In addition, some coumarins are also found in microorganisms, for example, in antibiotics such as novobiocin, coumermycin A1, and chlorobiocin [3,4]. Coumarin derivatives are typically synthesized by chemical modification of the coumarin ring. Owing to their diverse pharmacological properties and natural sources of origin, coumarins play an important role in the synthesis of natural products [5,6,7]. Furthermore, coumarins find widespread applications in a broad range of fields, including foods, cosmetics, as dispersive fluorescent laser dyes, as light-activated compounds in the field of medicine, and as anticoagulants in the production of pesticides [8]. Recently, several improved synthetic methodologies have been developed that use a variety of Lewis acid catalysts [9,10,11,12], phase transfer catalysts [13,14,15,16,17], microwave reactions [18], and molecular iodine [19]. Some of these methods are expensive, environmentally unfriendly, produce low yields, are incompatible with other functional groups, and involve labor-intensive product isolation procedures. Thus far, several methods, including Perkin [20], Knoevenagel [21], Reformatsky [22], Wittig [23], and Pechmann [24] reactions, have been adopted for the synthesis of coumarins. Therefore, a simple, efficient, and green chemistry for one-pot coumarins synthesis under mild conditions is required. The method presented herein involves the condensation of phenols with β-ketoesters, often in the presence of acid, which acts as a catalyst for the synthesis of coumarins. The superiority of use of FeF_3_ to the current process is demonstrated in comparison with other Lewis acids, Fe-salts, fluoride sources and insights into the origin of the efficiency are discussed [25,26].

**Scheme 1:**
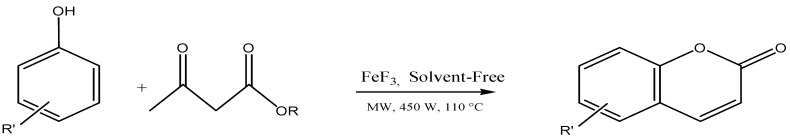
FeF_3_ catalyzed Pechmann reaction.

Previously, we have synthesized a number of heterocyclic compounds [27,28,29,30,31,32,33,34,35,36]. In this study, we have used of analyzed the Pechmann reaction to develop a new and suitable methodology for the synthesis of coumarins. The experiments were started with the study of one-pot, two-component Pechmann condensation using FeF_3_ as a catalyst under solvent-free microwave irradiation (Scheme 1).

## 2. Results and Discussion

Coumarins occupy an important place in the realm of natural products and synthetic organic chemistry. Coumarins are simple heterocyclic compounds that can be obtained from natural sources, especially green plants. They are used in food additives, perfumes, cigarettes, cosmetics, pharmaceuticals, light-activated compounds, and fluorescent laser dyes.

**Table 1:** FeF_3_ catalyzed synthesis of coumarin derivatives ^a^.

Entry	Phenol	Product	Time (min)	Yield (%)	MP °C, (Lit) [ref.]
1	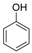	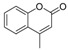	8	97	80–82, (81) [37]
2	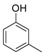	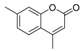	9	98	132–135, (131–133) [9]
3	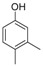	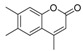	9	93	172–174, (171–172) [9]
4	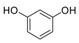	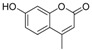	7	95	185–188, (184–185) [9]
5	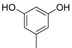	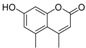	7	94	258–260, (257–260) [38]
6	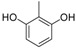	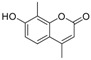	8	89	135–138, (137–138) [37]
7	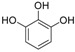	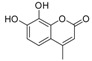	7	90	235–236, (234–237) [37]
8	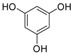	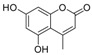	6	92	281–284, (280–281) [9]
9	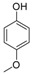	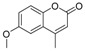	6	93	165–170, (169–170) [9]
10	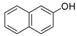	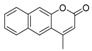	7	87	176–180, (180–182) [9]
11	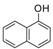	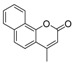	8	85	153–156, (154–155) [9]
12	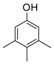	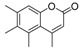	9	61	165–169
13	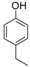	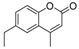	8	71	160–162
14	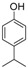	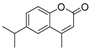	9	66	168–170

^a^ Reaction conditions: phenols (1 mmol), ethyl acetoacetate(1 mmol), FeF_3_ (0.05 g), Isolated yield.

In this research, we have synthesized some coumarins derivatives using phenols and ethyl acetoacetate in the presence of FeF_3_ as a catalyst to create the corresponding products, as illustrated in the model reaction (Scheme 1). The synthesis of compound **4** was selected as the model to optimize the reaction conditions. The corresponding results are summarized in Table 1. As can be seen from the results presented in this table, FeF_3_ acts as an effective catalyst, significantly increasing the reaction rate; moreover, it can be easily separated (Table 1). All the reactions were monitored by using thin layer chromatography (TLC) and carried forward to maximum atom utilization. In addition, all the products were characterized by using melting points, infrared spectroscopy (IR), proton nuclear magnetic resonance spectroscopy (^1^H-NMR), carbon-13 nuclear magnetic resonance spectroscopy (^13^C-NMR), mass spectroscopy and carbon, hydrogen, and nitrogen analysis (CHN). The results obtained from these systematic analysis were found to be in good agreement to those reported in the literature.

We have also carried out the model reaction under microwaves using different powers, and it was found that if the reactions are carried out without microwave irradiation they takes more time (60 min) and give negligible yields (26%). As the power increases (100, 250, 300, 450, 600 W), there is increase in yield with a corresponding decrease in reaction time up to 450 W, but no significant change is observed at 600 W. Hence, we selected 450 W at 110 °C and 1 atm pressure for all the subsequent reactions. The different reaction conditions obtained by varying the amount of catalyst and the corresponding results are summarized in Table 2. It could be observed that the product yield is strongly affected by the amount of catalyst used in the reaction. Best results were obtained in under solvent-free microwave irradiation (Entry 4) using 0.05 g of catalyst.

**Table 2:** FeF_3_ catalyzed synthesis of 7-hydroxy-4-methyl-chromen-2-one (**4**) in various amount of the catalyst under solvent-free microwave irradiation ^a^.

NO.	Catalyst (g)	Yield (%)
1	-	15
2	0.02	69
3	0.04	86
4	0.05	95
5	0.06	91
6	0.07	89
7	0.08	87
8	0.10	85

^a^ Reaction conditions: resorsinol (1 mmol), ethyl acetoacetate (1 mmol), and catalyst at 7 min; Isolated yield.

To compare the efficiency of the solvent-free *versus* solution conditions, the reaction was examined in several solvents and solvent-free under microwave irradiation. Thus, a mixture of resorsinol (1 mmol), ethyl acetoacetate (1 mmol), and FeF_3_ (0.05 g) was heated under microwave irradiation for 7 min in different solvents. The results are listed in Table 3. As it is clear from the results, lower yields and longer reaction times were observed under solution conditions. Therefore, the solvent-free methid offers the as a best and more efficient conditions.

**Table 3:** FeF_3_ catalyzed synthesis of 7-hydroxy-4-methyl-chromen-2-one (**4**) under various solvent and solvent-free conditions.

NO.	Solvent	Yield (%)
1	solvent-free	95
2	DMF	57
3	acetonitrile	63
4	dichloromethane	49
5	water	64
6	ethanol	81
7	methanol	80
8	dioxane	74

Comparison of reaction conditions and product yield between previously reported methods and the reaction of resorcinol with ethyl acetoacetate (Table 1, Entry 4) in the presence of different catalysts is shown in Table 4. The catalyst was easily recovered by simple filtration after dilution of the reaction mixture with ethyl acetate and was reused after being vacuum dried. FeF_3_ was reused for four runs without significant loss of activity (Run 1: 95%; Run 2: 92%; Run 3: 89%; Run 4: 87%; Run 5: 82%).

**Table 4:** Reaction of resorcinol with ethyl acetoacetate (Table 1, Entry 4) in the presence of different catalysts.

Entry	Catalyst/mol%	Conditions	Reaction Time (min)	Yield (%)	Reference
1	Ce(OTf)4/1	H_2_O/Room Temperature	15	92	[39]
2	PFPAT/10	Toluene/110 °C	180	90	[39]
3	MFRH/0.05 g	Solvent free/80 °C	50	65	[39]
4	Oxalic acid/10	Solvent free/80 °C	30	95	[39]
	Nanoreactors/7	Solvent free/130 °C	60	30	[39]
**5**	**FeF_3_/0.05 g**	**Heating/Ethanol, reflux**	**120**	**67**	**This Research**
**5**	**FeF_3_/0.05 g**	**Microwaves**	**7**	**95**	**This Research**

All the title compounds **1**–**14** were screened for their antimicrobial activity. They were first screened for anti-bacterial activity against the growth of *Staphylococcus aureus* (Gram + ve) and *Escherichia coli* (Gram − ve) at different concentrations (100, 50, 25 ppm) by the disk diffusion method. All the compounds show good activity against both bacteria when compared to the reference compound penicillin. Then next they were subjected to antifungal activity evaluation against the growth of *Aspergillus niger* and *Helminthosporium oryzae* at various concentrations (100, 50, 25 ppm) with griseofulvin as the standard reference compound. The inhibition zone results of title compounds were presented in Table 5. The majority of the compounds showed good antifungal activity against both fungi, especially compounds **3**, **7**, **10** and **12**.

**Table 5:** Antimicrobial activity of the compounds **1**–**14** (µg/mL).

Compound	Zone of Inhibition (%)											
	Antibacterial Activity						Antifungal Activity					
	*Escherichia coli*			*Staphylococcus aureus*			*Aspergillus niger*			*Helminthosporium oryzae*		
	100	50	25	100	50	25	100	50	25	100	50	25
1	21	10	5	22	10	6	20	10	7	13	8	5
2	22	11	6	21	10	5	18	12	6	19	12	7
3	23	12	7	23	12	6	19	13	8	13	7	4
4	20	12	7	21	10	5	21	11	7	19	10	7
5	22	11	6	21	12	6	18	11	6	13	7	4
6	22	10	6	22	12	7	19	9	4	18	10	5
7	24	14	8	23	12	7	21	14	8	20	15	8
8	21	10	5	22	11	5	20	12	4	14	9	5
9	21	11	7	21	12	5	20	12	6	19	10	7
10	23	12	8	23	10	6	21	13	6	20	11	6
11	21	10	5	20	10	6	20	10	5	19	11	6
12	23	13	7	23	12	8	20	12	7	20	10	5
13	22	10	5	21	10	6	19	11	5	16	8	6
14	22	11	5	22	10	5	20	11	4	15	9	5
Penicillin Griseofulvin	20	12	8	20	12	8	20	10	5	20	10	5

## 3. Experimental Section

### 3.1. General Information

Melting points were measured on an Electrothermal 9100 apparatus. All reactions were carried out in a CEM MARS 5^TM^ microwave oven. The TLC was performed with silica gel SILG/UV 254 plates. IR spectra were measured using a Shimadzu IR-470 spectrophotometer. ^1^H- and ^13^C-NMR spectra were determined on a Bruker 400 DRX AVANCE instrument at 400 and 100 MHz, respectively. The elemental analyses (C, H) were conducted using Carlo ERBA Model EA 1108 and Perkin-Elmer 240c analyzers. Mass spectra were recorded on a Jeol JMSD-400 spectrometer.

### 3.2. Typical Procedure Adopted for the Synthesis of 7-Hydroxy-4-Methyl-Chromen-2-One (**4**)

A mixture of resorsinol (1 mmol), ethyl acetoacetate (1 mmol), and FeF_3_ (0.05 g) was ground in an open Pyrex beaker and the homogenized mixture was heated by microwave irradiation for about 7 min, as indicated in Table 1. The progress of the reaction was monitored by using TLC (ethyl acetate/*n*-hexane: 1/2). After complete conversion as indicated by TLC, the mixture was extracted with petroleum ether (3 × 30 mL) and washed with water (3 × 30 mL). The crude products were purified by recrystallization from ethanol (95%) to afford pure products. Data for new compounds are listed below:

*4,5,6,7-Tetramethyl-2H-chromen-2-one* (**12**): Yellow solid; m.p.: 165–169 °C; IR (KBr) ν_max_ (cm^−1^): 1674 (ester C=O stretch), 1602 (C−C=C stretch); ^1^H-NMR (DMSO-*d*_6_) δ: 2.12 (s, 3H, C*H_3_*), 2.20 (s, 3H, C*H_3_*), 2.31 (s, 3H, C*H_3_*), 2.42 (s, 3H, C*H_3_*), 6.25 (m, 1H), 7.24 (s, 1H); ^13^C-NMR (DMSO-*d*_6_) δ: 14.4, 17.6, 21.8, 23.7, 114.5, 117.0, 121.7, 125.6, 131.8, 133.7, 152.1, 158.8. MS (*m/z*): 202 (M+); Anal. Calcd for C_13_H_14_O_2_: C, 77.30; H, 7.03%. %. Found: C, 77.15; H, 6.86%.

*6-Ethyl-4-methyl-2H-chromen-2-*one (**13**): Yellow solid. m.p.: 160–162 °C. IR (KBr) ν_max_ (cm^−1^): 1666 (ester C=O stretch), 1589 (C−C=C stretch). ^1^H-NMR (DMSO-*d*_6_) δ: 1.85 (t, *J* = 7.2, 3H, C*H_3_*), 2.38 (s, 3H, C*H_3_*), 3.63 (q, *J* = 7.2, 2H, C*H_2_*), 5.91 (m, 1H), 7.11 (d, *J* = 8.5, 1H), 7.34 (dd, *J* = 8.5, 2.2, 1H), 7.41 (s (br), 1H); ^13^C-NMR (DMSO-*d*_6_) δ: 16.8, 18.8, 22.4, 115.4, 118.6, 123.3, 124.8, 130.9, 135.1, 155.3, 161.2. MS (*m/z*): 188 (M+). Anal. Calcd for C_12_H_12_O_2_: C, 76.57; H, 6.43%. Found: C, 77.25; H, 6.38%.

*6-Isopropyl-4-methyl-2H-chromen-2-*one (**14**): Yellow solid; m.p.: 168–170 °C. IR (KBr) ν_max_ (cm^−1^): 1658 (ester C=O stretch), 1585 (C−C=C stretch). ^1^H-NMR (DMSO-*d*_6_) δ: 1.65 (d, *J* = 6.5, 1H, C*H*), 2.44 (s, 3H, C*H_3_*), 2.95 (q, *J* = 6.5, 6H, 2C*H_3_*), 5.89 (m, 1H), 7.15 (d, *J* = 8.3, 1H), 7.30 (dd, *J* = 8.3, 2.3, 1H), 7.45 (s (br), 1H); ^13^C-NMR (DMSO-*d*_6_) δ: 14.8, 15.6, 18.5, 24.9, 116.7, 119.2, 122.7, 126.9, 133.4, 137.3, 157.4, 163.5. MS (*m/z*): 202 (M+). Anal. Calcd for C_13_H_14_O_2_: C, 77.20; H, 6.98%. Found: C, 76.55; H, 6.68%.

### 3.3. Antimicrobial Activity

**Figure 1:**
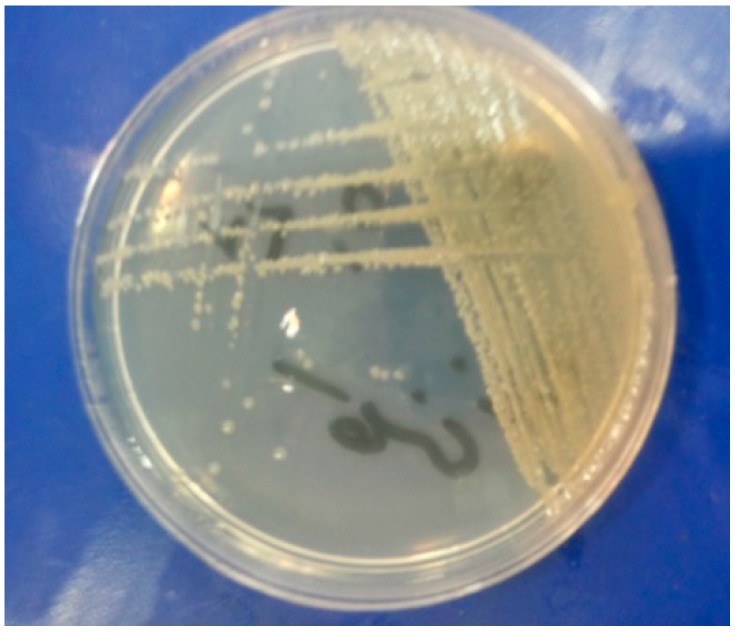
*Escherichia coli*.

**Figure 2:**
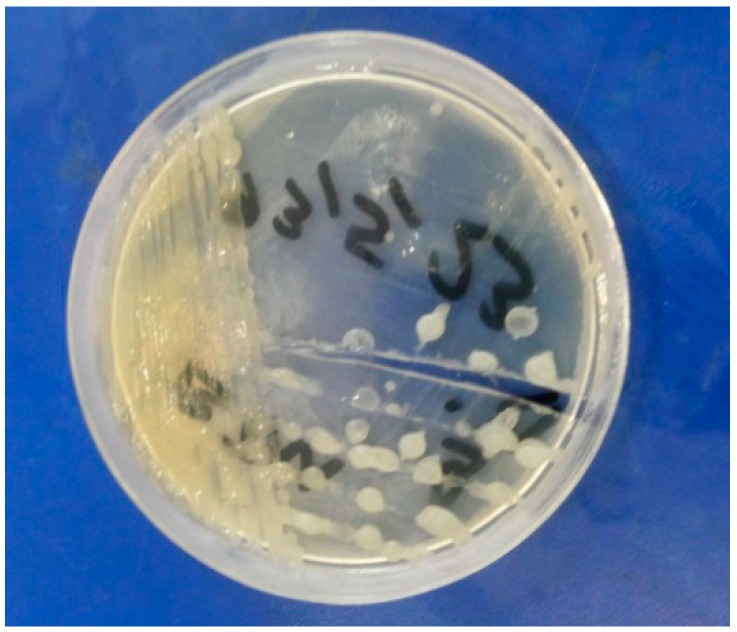
*Staphylococcus aureus*.

**Figure 3:**
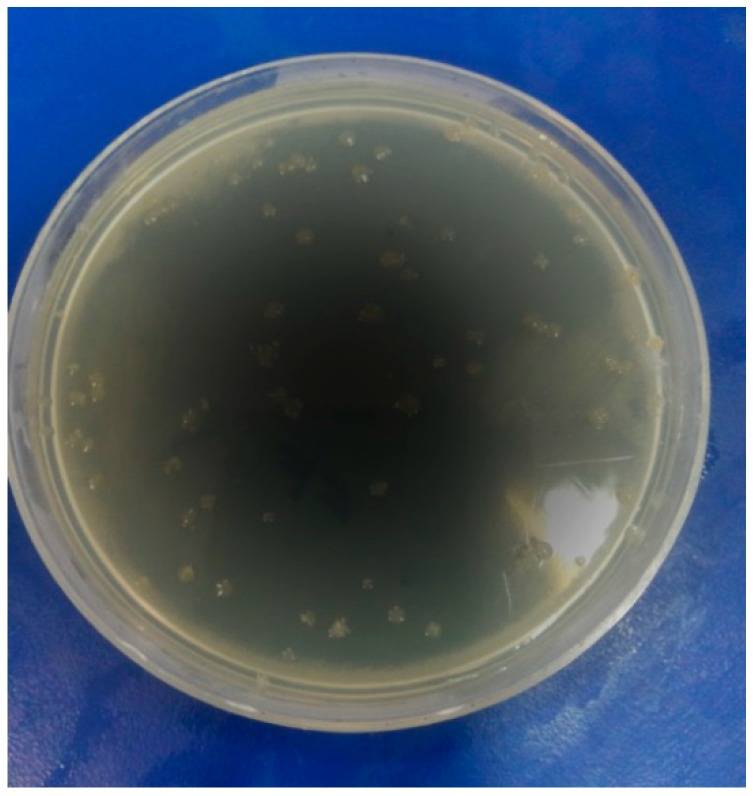
*Aspergillus niger*.

**Figure 4:**
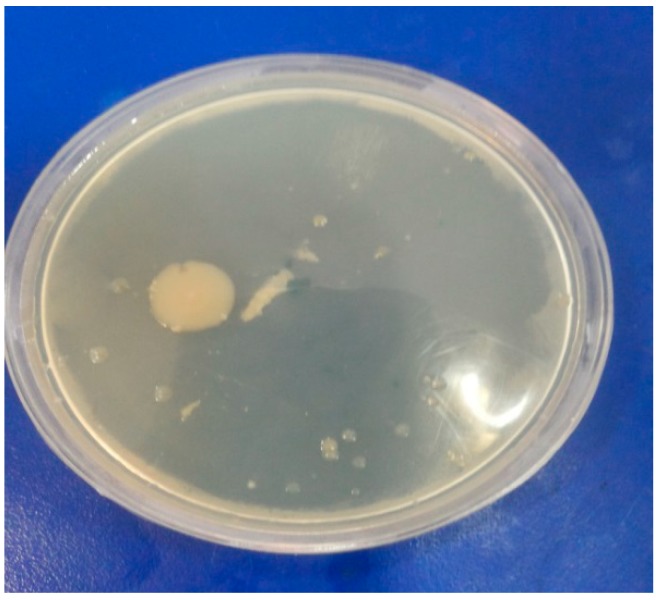
*Helminthosporium oryzae*.

The compounds **1**–**14** were screened by the disk diffusion method [40,41], for their antimicrobial activity against the bacteria *Escherichia coli* and *Staphylococcus aureus* and fungi *Aspergillus niger* and *Helminthosporium oryzae* by comparison with the standard bactericide penicillin and standard fungicide griseofulvin at three different concentrations (100, 50, 25 ppm). The tubes were incubated aerobically at 37 °C for 18–24 h. The experiments were run in triplicate and the average results are reported in Table 4. *Escherichia coli*, *Staphylococcus aureus*, *Aspergillus niger* and *Helminthosporium oryzae* are shown in Figure 1, Figure 2, Figure 3 and Figure 4.

## 4. Conclusions

In summary, we have demonstrated a novel methodology based on the Pechmann condensation for the synthesis of substituted coumarins under solvent-free microwave irradiation conditions, catalyzed by FeF_3_ as an effective eco-friendly catalyst. Moderate to high yields of the corresponding coumarins were obtained. The unique advantages of this method include a one-pot synthesis strategy, experimental simplicity under solvent-free microwave irradiation, high yields obtained under short reaction times, easy and quick isolation of the products. The majority of the compounds **1**–**14** exhibited significant activity against selected bacteria and fungi with inhibition zones almost comparable to those of the standard drugs. Thus a new group of compounds with comparable antimicrobial potency to some presently used commercial bactericides/fungicides has been discovered.
